# Does retirement mean more physical activity? A longitudinal study

**DOI:** 10.1186/s12889-016-3253-0

**Published:** 2016-07-20

**Authors:** Xiaoqi Feng, Karen Croteau, Gregory S. Kolt, Thomas Astell-Burt

**Affiliations:** Early Start Research Institute, University of Wollongong, Northfields Ave, Wollongong, NSW 2522 Australia; School of Health and Society, University of Wollongong, Northfields Ave, Wollongong, 2522 NSW Australia; Illawarra Health and Medical Research Institute, Northfields Ave, Wollongong, 2522 NSW Australia; St Joseph’s College of Maine, Standish, 04084 Maine USA; School of Science and Health, Western Sydney University, Locked Bag 1797, Penrith, NSW 2751 Australia

**Keywords:** Retirement, Physical activity, Aging

## Abstract

**Background:**

Evidence on physical activity (PA) and transitions out of full-time employment in middle-to-older age is mainly cross-sectional and focused upon retirement. The purpose was to examine trajectories in PA before and after transitions out of full-time employment.

**Methods:**

Data were obtained for 5,754 people in full-time employment aged 50–75 from the US Health and Retirement Survey. Logistic regression was used to examine trajectories in twice-weekly participation in light, moderate and vigorous PA among those transitioning to part-time work, semi-retirement, full retirement, or economic inactivity due to disability, in comparison to those remaining in full-time employment.

**Results:**

Twice weekly participation in vigorous and light physical activity changed little for those who remained in full-time employment, while moderate physical activity decreased between baseline and follow-up (OR 0.95, 95 % CI 0.91, 0.99). Differences in physical activity according to transitional categories at follow-up were evident. Baseline differences in physical activity across all intensities were greatest among participants transitioning from full-time to part-time employment compared to those who remained in full-time employment throughout the study period (vigorous OR 1.41 95 % CI 1.23, 1.61; moderate OR 1.28 95 % CI 1.12, 1.46; light OR 1.29 95 % CI 1.12, 1.49). Those transitioning to unemployment were already among the least physically active at baseline, irrespective of intensity (albeit, with 95 % CIs spanning unity). Those transitioning to full-time retirement were also among the least active (e.g. vigorous OR 0.71 95 % CI 0.61, 0.81; moderate OR 0.80 95 % CI 0.71, 0.90). Declines in physical activity were reported for those transitioning to economic inactivity due to a disability (vigorous OR 0.29 95 % CI 0.14, 0.64; moderate OR 0.56 95 % CI 0.33, 0.95; light OR 0.34 95 % CI 0.19, 0.63). Physical activity increased regardless of intensity among participants transitioning to semi-retirement (*p* > 0.05) and full retirement (e.g. vigorous OR 1.28 95 % CI 1.09, 1.51; moderate OR 1.24 95 % CI 1.07, 1.43). Light physical activity increased for those transitioning to unemployment (OR 1.40 95 % CI 1.02, 1.93), though less change was evident in moderate or vigorous physical activity.

**Conclusions:**

The amount and intensity of PA varies by the type of transition out of full-time employment among people in middle-to-older age.

## Background

Participation in physical activity declines with age [1] concurrent to an increasing risk of preventable health conditions like type 2 diabetes [2, 3]. Yet physical activity is widely recognized as crucial for strengthening and maintaining physical and mental health during aging [4, 5]. Some transitions out of the labor market, such as retirement and semi-retirement, may free up time that could be used to (re) engage in physical activity. Retirement can be seen, therefore, as a potentially sensitive period in the lifecourse to target interventions for promoting healthy ageing [6, 7].

Evidence on physical activity during retirement from cross-sectional studies is mixed [8–10] and limited by the spectre of reverse causality. Some longitudinal studies have the potential to approximate the *transition* to retirement, so should be regarded as higher quality evidence [11–18]. Of the longitudinal studies, some have attempted to isolate the impact of retirement on leisure-time physical activity specifically (e.g. [12, 16]). Others have investigated whether trajectories in physical activity across retirement vary by indicators of socioeconomic circumstances (e.g. [11]). Findings remain equivocal, however, providing no firm answer on how retirement affects participation in physical activity.

Importantly, most studies focus upon the transition from employment to retirement *per se*, but differences in physical activity are likely between full retirees and those who retain a level of part-time employment, semi-retired, or become economically inactive due to disability. These differences may not only manifest in terms of frequency of participation, but also in terms of how intense the physical activity is. Prior research has shown that higher intensity physical activity (e.g. jogging) accrues more health benefits than less vigorous forms of recreation (e.g. gentle swimming). It is unknown to what extent transitions out of the labor market influence participation in different intensities of physical activity, but this knowledge is of public health interest.

Accordingly, the purpose of this longitudinal study was to examine participation in different intensities of physical activity among people transitioning out of full-time employment to different forms of retirement, while also accounting for transitions to unemployment, part-time work, or disability status.

## Method

### Data

Data on physical activity among people transitioning out of full-time employment aged 50 to 70 years were extracted from the US Health and Retirement Survey (HRS) [19]. The HRS is a representative source of longitudinal data collected bi-annually, with initial surveys conducted in each member’s home and follow-up interviews mainly by telephone. A multi-stage area probability sample design was implemented with four distinct selection stages. The first stage of sampling involved probability proportionate to size selection of US Metropolitan Statistical Areas (MSAs) and non-MSA counties. Second, area segments within each of the sampled MSA and non-MSA counties were selected. Third, a systematic selection of housing units were obtained from a complete listing of all housing units physically located within each area segment. Finally, housing units were selected. Oversamples were obtained of Blacks, Hispanics and residents of the state of Florida. Further details on sampling are available online [19]. Participation rates in follow-up surveys were very high (between 92 and 95 %). The HRS was approved by the University of Michigan’s Health Sciences Human Subjects Committee, sponsored by the National Institute on Aging (grant number NIA U01AG009740) and is conducted by the University of Michigan. Our study made use of the de-identified publically available data file prepared by the RAND Center for the Study of Aging.

### Sample

**Fig. 1:**
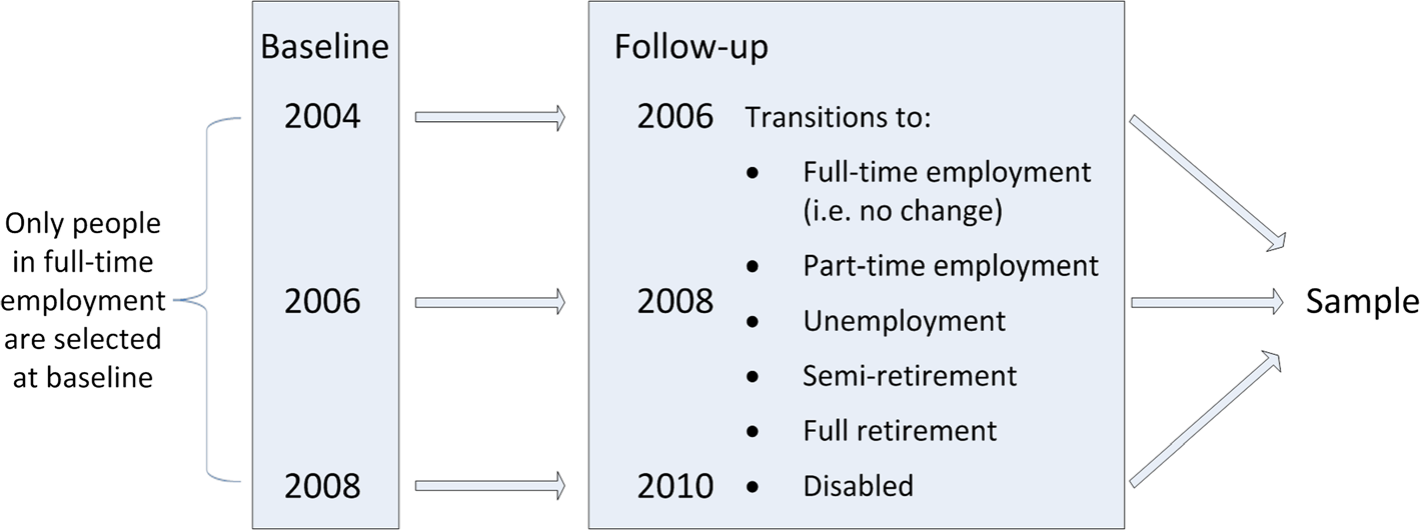
Sampling strategy

Transition out of full-time employment and potential change in physical activity was assessed using a pre-post design with survey responses from participants in full-time employment in 2004, 2006 and 2008 hereafter referred to as ‘baseline’ (Fig. 1). The sample was restricted to participants aged 50 to 70, spanning the national retirement age of 65y. Participants could contribute to multiple waves of data collection. This resulted in an overall sample of 5,754 people and 24,224 person-years (the total number of observations in the sample at baseline and follow-up).

### Physical activity

Baseline members appearing in the consecutive survey (i.e. 2004 to 2006, 2006 to 2008, 2008 to 2010) were asked identical questions on leisure-time and work-related ‘vigorous’, ‘moderate’, and ‘light’ physical activity. Participants were able to respond: everyday, more than once per week, once per week, one to three times per month, or never. The wording of each question was as follows:

- *“How often do you take part in sports or activities that are vigorous, such as running or jogging, swimming, cycling, aerobics or gym workout, tennis, or digging with a spade or shovel?”* (classified by the authors as vigorous physical activity).
- *“And how often do you take part in sports or activities that are moderately energetic such as, gardening, cleaning the car, walking at a moderate pace, dancing, floor or stretching exercises?”* (classified by the authors as moderate physical activity).
- *“And how often do you take part in sports or activities that are mildly energetic, such as vacuuming, laundry, home repairs?”* (classified by the authors as light physical activity).

Binary variables for each measure were created, with the cut-point set at twice per week versus fewer occasions. This cut-point was a pragmatic choice standardized across vigorous, moderate and light intensities of physical activity, since responses more closely aligning with published physical activity guidelines [20] were not attainable.

### Transitions out of full-time employment

Participants self-reported evidence of working, being retired, and disability. HRS considers working full-time as 35+ hours per week, at 36+ weeks per year. Less than 35 h per week is classified as part-time. HRS classifies people as semi-retired if they reported being part-time workers along with a mention of retirement. For people who were looking for work and did not mention retirement, they were classified as unemployed regardless of their age. Those mentioning retirement, who were not employed and not looking for work were classified as fully retired. All non-working people reporting a disability with no mention of retirement were classified as disabled. People who did not report any of the above were classified as ‘not in the labor force’. A categorical variable was created describing transitions out of full-time employment at follow-up for every participant, including those who remained in full-time employment.

### Adjustment for confounding

Participation in physical activity is well known to decline with age, with effect measure modification of this association by gender and potentially also by cohort [21]. Time and status in the labor market, the risk of disability and unemployment are similarly related to age, gender and cohort [22, 23]. As such, participants’ age, gender and the baseline year of survey were included in the models to address potential sources of confounding.

### Statistical analysis

The odds of participating in light, moderate or vigorous physical activity at least twice a week in relation to transitions out of full-time employment were estimated using logistic regression. Transitions out of full-time employment were fitted as categories, with people who remained in full-time employment as the reference group. Time was addressed using a binary variable and an interaction was fitted with transition to allow physical activity to vary accordingly. Controls for age, gender and cohort were introduced sequentially. Robust standard errors were used to adjust for repeated measures of the same participants over time [24]. Parameter estimates for logistic regression were exponentiated to Odds Ratios (OR) and 95 % Confidence Intervals (95 % CI). Analyses were conducted in 2013.

## Results

### Descriptive results

Table 1 reports each transition group pooled across all cohorts (2004, 2006 and 2008) and time periods (baseline and follow-up). From a total of 23,842 person years, just over two-thirds remained in full-time employment. Approximately 11.8 % transitioned into part-time work, 10.2 % retired fully, and 7.1 % semi-retired, 2.2 % became unemployed and 0.7 % were classified as disabled. The mean age for those retiring (semi and fully) was just over 62 years old, in contrast to the mean age of all other transitional groups being under 60 years. The gender distribution among the groups remaining in full-time employment and becoming unemployed were almost equal. Conversely, there was a 4:1 ratio of women to men transitioning to part-time work. Women were also over-represented, though to a lesser degree, among those groups transitioning to semi-retirement, full retirement, and disability status. Participation in physical activity everyday was rare across each transitional group, regardless of intensity. The majority of participants reported being physically active at least twice per week, with some variation between groups and intensity. Non-participation in all intensities of physical activity, but vigorous physical activity in particular, was especially high among people moving out of full-time employment into disability status.

**Table 1:** Descriptive statistics, by transitions out of full-time employment

	Work full-time (no change)		Work part-time		Unemployed		Semi-retired		Fully retired		Disabled	
N person-years (%)	16,070	66.3	2,868	11.8	538	2.2	1,718	7.1	2,478	10.2	170	0.7
Vigorous physical activity (N, %)												
Never	7,743	48.2	1,310	45.7	276	51.3	839	48.8	1,386	55.9	122	71.8
1–3 times per month	1,756	10.9	272	9.5	50	9.3	181	10.5	211	8.5	6	3.5
Once per week	1,856	11.6	349	12.2	63	11.7	163	9.5	263	10.6	11	6.5
At least twice per week	4,215	26.2	855	29.8	133	24.7	490	28.5	556	22.4	29	17.1
Everyday	500	3.1	82	2.9	16	3.0	45	2.6	62	2.5	2	1.2
Moderate physical activity (N, %)												
Never	1,750	10.9	293	10.2	75	13.9	200	11.6	428	17.3	52	30.6
1–3 times per month	1,727	10.8	283	9.9	68	12.6	181	10.5	279	11.3	13	7.7
Once per week	2,972	18.5	454	15.8	106	19.7	307	17.9	412	16.6	25	14.7
At least twice per week	8,185	50.9	1,579	55.1	245	45.5	873	50.8	1,150	46.4	68	40.0
Everyday	1,436	8.9	259	9.0	44	8.2	157	9.1	209	8.4	12	7.1
Light physical activity (N, %)												
Never	685	4.3	87	3.0	26	4.8	80	4.7	167	6.7	31	18.2
1–3 times per month	981	6.1	104	3.6	39	7.3	106	6.2	164	6.6	10	5.9
Once per week	3,597	22.4	495	17.3	113	21.0	385	22.4	534	21.6	34	20.0
At least twice per week	9,056	56.4	1,805	62.9	291	54.1	937	54.5	1,359	54.8	80	47.1
Everyday	1,751	10.9	377	13.2	69	12.8	210	12.2	254	10.3	15	8.8
Age (Mean years, SD)	58.3	4.7	58.8	5.1	57.5	4.2	62.2	4.7	62.1	4.4	57.5	4.4
Gender (N, %)												
Male	8206	51.1	664	23.2	274	50.9	754	43.9	1130	45.6	68	40.0
Female	7864	48.9	2,204	76.9	264	49.1	964	56.1	1348	54.4	102	60.0

### Transition out of full-time employment

Table 2 reports findings from the logistic regressions. Twice weekly participation in vigorous and light physical activity changed little for those who remained in full-time employment, while moderate physical activity decreased between baseline and follow-up (OR 0.95, 95 % CI 0.91, 0.99). Differences in physical activity according to transitional categories at follow-up were evident. Baseline differences in physical activity across all intensities were greatest among participants transitioning from full-time to part-time employment compared to those who remained in full-time employment throughout the study period (vigorous OR 1.41 95 % CI 1.23, 1.61; moderate OR 1.28 95 % CI 1.12, 1.46; light OR 1.29 95 % CI 1.12, 1.49).

**Table 2:** Association between the propensity for vigorous, moderate and light physical activity at least twice a week, and transitions out of full-time employment (people who remained in full-time employment are the reference group): partially and fully adjusted logistic regression with robust standard errors

	Vigorous physical activity				Moderate physical activity				Light physical activity			
	Model 1		Model 2		Model 1		Model 2		Model 1		Model 2	
	OR	95 % CI	OR	95 % CI	OR	95 % CI	OR	95 % CI	OR	95 % CI	OR	95 % CI
Time (ref: baseline)												
Follow-up	0.94	(0.90, 0.98)***	0.99	(0.94, 1.03)	0.91	(0.88, 0.95)***	0.95	(0.91, 0.99)**	0.91	(0.88, 0.95)***	0.98	(0.94, 1.03)
Transition (ref: Work Full-Time)												
Work Part-Time	1.20	(1.05, 1.37)**	1.41	(1.23, 1.61)***	1.21	(1.06, 1.38)**	1.28	(1.12, 1.46)***	1.51	(1.31, 1.74)***	1.29	(1.12, 1.49)***
Unemployment	0.89	(0.68, 1.17)	0.86	(0.65, 1.14)	0.79	(0.61, 1.01)	0.77	(0.60, 0.99)*	0.85	(0.66, 1.09)	0.81	(0.63, 1.05)
Semi-Retired	1.01	(0.86, 1.18)	1.15	(0.98, 1.35)	0.96	(0.83, 1.11)	1.05	(0.90, 1.21)	0.94	(0.81, 1.10)	1.05	(0.90, 1.22)
Fully Retired	0.71	(0.61, 0.81)***	0.79	(0.69, 0.92)**	0.74	(0.65, 0.83)***	0.80	(0.71, 0.90)***	0.87	(0.77, 0.99)*	0.96	(0.85, 1.10)
Disabled	0.86	(0.53, 1.40)	0.90	(0.55, 1.46)	0.79	(0.51, 1.21)	0.79	(0.51, 1.21)	1.05	(0.66, 1.67)	0.95	(0.59, 1.53)
Transition x Time												
Time x Work Part-Time	0.95	(0.85, 1.07)	0.95	(0.85, 1.07)	0.99	(0.88, 1.11)	0.99	(0.88, 1.11)	1.06	(0.92, 1.21)	1.06	(0.92, 1.22)
Time x Unemployment	1.09	(0.80, 1.48)	1.09	(0.80, 1.48)	1.02	(0.77, 1.34)	1.02	(0.77, 1.34)	1.39	(1.02, 1.89)*	1.40	(1.02, 1.93)*
Time x Semi-Retired	1.17	(0.99, 1.38)	1.17	(0.99, 1.39)	1.11	(0.94, 1.30)	1.11	(0.94, 1.30)	1.08	(0.90, 1.29)	1.08	(0.90, 1.30)
Time x Fully-Retired	1.28	(1.09, 1.50)**	1.28	(1.09, 1.51)**	1.24	(1.07, 1.43)**	1.24	(1.07, 1.43)**	1.10	(0.95, 1.28)	1.11	(0.95, 1.29)
Time x Disabled	0.30	(0.14, 0.64)**	0.29	(0.14, 0.64)**	0.56	(0.33, 0.95)*	0.56	(0.33, 0.95)*	0.35	(0.20, 0.64)***	0.34	(0.19, 0.63)***
Cohort (ref: 2004)												
2006	0.97	(0.92, 1.01)	0.99	(0.94, 1.04)	0.99	(0.95, 1.04)	1.01	(0.96, 1.05)	1.01	(0.96, 1.06)	1.03	(0.98, 1.09)
2008	0.97	(0.91, 1.04)	1.02	(0.95, 1.09)	0.90	(0.85, 0.96)***	0.93	(0.87, 0.99)*	0.95	(0.89, 1.01)	0.98	(0.91, 1.05)
Gender (ref: Male)												
Female	-		0.60	(0.54, 0.66)***	-		0.84	(0.77, 0.91)***	-		1.92	(1.76, 2.09)***
Age	-		0.98	(0.97, 0.99)***	-		0.98	(0.97, 0.99)***	-		0.96	(0.95, 0.97)***
N (Observations)	24224		24224		24224		24224		24224		24224	
N (Clusters)	5754		5754		5754		5754		5754		5754	

*** *p* < 0.001; ** *p* < 0.01; * *p* < 0.05

### Unemployment, economic inactivity due to disability and (semi) retirement

Table 2 also shows those transitioning to unemployment were already among the least physically active at baseline irrespective of intensity (albeit, with 95 % CIs spanning unity). Those transitioning to full-time retirement were also among the least active (e.g. vigorous OR 0.71 95 % CI 0.61, 0.81; moderate OR 0.80 95 % CI 0.71, 0.90). Declines in physical activity were reported for those transitioning to economic inactivity due to a disability (vigorous OR 0.29 95 % CI 0.14, 0.64; moderate OR 0.56 95 % CI 0.33, 0.95; light OR 0.34 95 % CI 0.19, 0.63). Physical activity increased regardless of intensity among participants transitioning to semi-retirement (*p* > 0.05) and full retirement (e.g. vigorous OR 1.28 95 % CI 1.09, 1.51; moderate OR 1.24 95 % CI 1.07, 1.43). Light physical activity increased for those transitioning to unemployment (OR 1.40 95 % CI 1.02, 1.93), though less change was evident in moderate or vigorous physical activity.

### Trajectories in physical activity

**Fig. 2:**
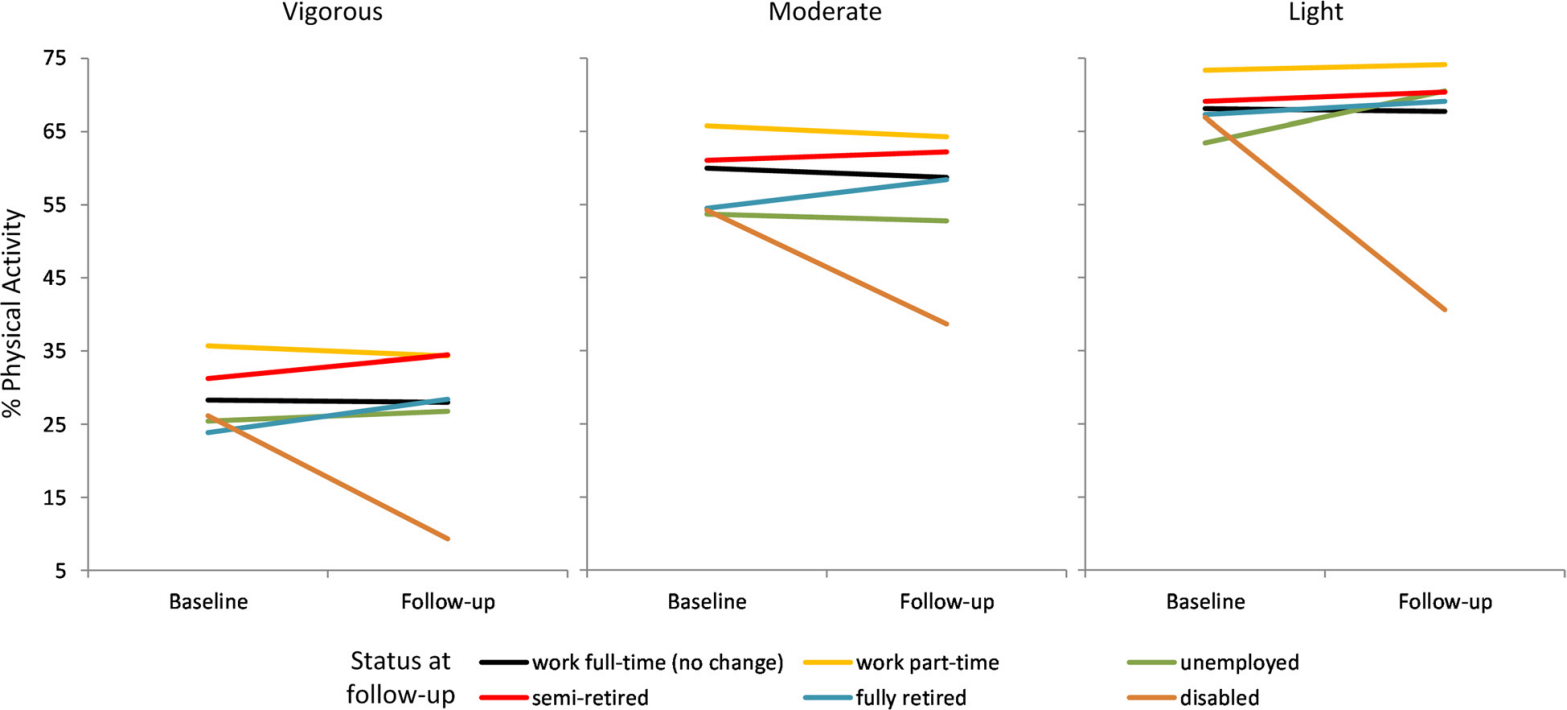
Vigorous, moderate and light physical activity at least twice a week (age and gender adjusted), by transitions out of full-time employment (people who remained in full-time employment are the reference group). *Adjusted for age, gender, cohort

Figure 2 depicts the aforementioned results visually to aid interpretation. The most visually striking finding is the decline in physical activity, regardless of intensity, among people transitioning out of full-time employment to disability. The rise in light physical activity among those becoming unemployed is evident, as is also the higher levels of physical activity at baseline among those moving from full- to part-time employment. Some increase in physical activity was also observable among participants transitioning into semi and full retirement.

## Discussion

Insufficient physical activity is suggested to cause 6 % of coronary heart disease, 7 % of type 2 diabetes, 10 % of breast cancer, 10 % of colon cancer, and 9 % of premature mortality [25]. Although finding opportunities to promote the initiation and maintenance of physically active lifestyles is needed across the lifecourse, this study supports previous evidence that indicates the process of retirement as one such time period [8–18].

Our findings enhance prior knowledge by revealing that participation in physical activity varies by the type of transition out of full-time employment among people approaching retirement. To indicate that there is merely one pathway to retirement does not acknowledge the complexity involved. The clear differences in physical activity among people transitioning from full-time employment to semi-retirees, full retirees, part-time workers and the unemployed among people in middle-to-older age is a potentially important finding that warrants attempts at replication.

Inevitably, the findings raise questions and hypotheses requiring analyses that are beyond the remit of the paper and, in some cases, also the data available. For example, is the rise in physical activity regardless of intensity among people moving into semi-retirement due to less time spent in employment? Why are people who move into part-time work already more physically active than their counterparts who remained in full-time work? Is the rise in light physical activity among people who become unemployed sustained among those who re-enter some level of employment? What factors buffer the potential impact of disability on the substantial decreases in physical activity? What types of activities do people become more or less engaged in and are there differences between transitional groups? To what extent do changes in physical activity coinciding with the transition out of full-time employment reflect personal choices versus any number of possible competing demands upon time, including informal caring and volunteering? This is not an exhaustive list and it is clear that much remains unknown. Yet, the need to promote physical activity in ageing populations remains a pressing concern and these hypotheses warrant investigation in order to target future interventions accordingly.

### Strengths and limitations

A merit of this study is the longitudinal design with which changes in physical activity can be observed in association with transitions out of full-time employment. Cross-sectional studies, by comparison, are unable to address this putative change in exposure without incurring bias. Variation in results by intensity of physical activity indicates the ability to differentiate between light, moderate and vigorous forms to be a further strength.

Data prior to 2004 could not be included as wording of physical activity questions varied from those from 2004 onwards, restricting the sample size. Furthermore, self-reporting of physical activity may be prone to error related to unmeasured factors that also determine transitions out of full-time employment. Objective measurement of physical activity was not possible in this case, but future work in this regard would help to further enhance a growing scientific literature on retirement and physical activity.

Overlap between transitional categories during the period between baseline and follow-up is somewhat inevitable and that additional complexity could influence the results in unpredictable ways. Many people, for example, may self-classify as disabled and retired, but in these analyses they would be in the retired category, with the disabled category reserved for those who were not retired. Disentangling trajectories in physical activity across more granular transitional categories may be possible in future research.

## Conclusion

Transitions out of full-time employment are heterogeneous. Trajectories in physical activity that coincide with these transitions are similarly variable. Further investigation is needed to replicate these findings and to determine potential reasons why, in order to identify potential points for intervention.

## Abbreviations

95 % CI, 95 % confidence intervals; HRS, health and retirement survey; MSA, Metropolitan Statistical Areas; NIA, National Institute on Aging; OR, odds ratios; PA, physical activity
